# 

**Published:** 1986-04

**Authors:** 


					MEDICO
CHIRURGICAL
JOURNAL
VOLUME 101 (ii)
APRIL 1986
The Imtran Revolution
Histopathological audit
Dr Robert Levet: an obscure practiser in physic
Gun Money?Owning a piece of history
MEDICINE
THE MEDICAL JOURNAL OF THE SOUTH WEST

				

